# A 235 Kb deletion at 17q21.33 encompassing the *COL1A1*, and two additional secondary copy number variants in an infant with type I osteogenesis imperfecta: A rare case report

**DOI:** 10.1002/mgg3.1241

**Published:** 2020-04-13

**Authors:** Numbereye Numbere, David R. Weber, George Porter, Mohammed A. Iqbal

**Affiliations:** ^1^ Department of Pathology and Laboratory Medicine University of Rochester Medical Center Rochester NY USA; ^2^ Pediatric Endocrinology Department of Pediatrics University of Rochester Medical Center Rochester NY USA; ^3^ Pediatric Cardiology Department of Pediatrics University of Rochester Medical Center Rochester NY USA

**Keywords:** aCGH, *COL1A1*, MACGH, microarray comparative genomic hybridization, osteogenesis imperfecta

## Abstract

**Background:**

Osteogenesis imperfecta (OI) is a rare group of disorders characterized by increased susceptibility to fractures due to genetically determined bone fragility. About 90% of cases are due to mutations in *COL1A1* (17q21.33) or *COL1A2* (7q21.3) resulting in quantitative or qualitative defects in type I collagen, a key structural constituent of bone. OI due to complete *COL1A1* deletion is rare.

**Methods:**

We present a case of OI type I in a Caucasian female referred at 10 months of age for investigation of multiple fractures associated with minimal or no known trauma, small stature, and blue sclera. Her father has four to five lifetime fractures, blue sclera, normal stature, and a 14.5 kilobase (kb) deletion of *COL1A1* detected by targeted array performed at an outside institution. Microarray comparative genomic hybridization was performed on the proband and all members of the family.

**Results:**

A previously unreported 235 kb deletion at 17q21.33 encompassing *COL1A1*,* ITGA3*,* PDK2*,* SGCA*, and *HILS1* was detected in the proband. Also identified in both the proband and sibling is a maternally inherited 283 kb gain at 8p21.3 encompassing *CSGALNACT1* and a 163 kb loss at 10q21.3 encompassing *CTNNA3*. Analysis in the father revealed the same size deletion at 17q21.33 as in the proband.

**Conclusion:**

Together with previously reported cases of *COL1A1* deletions, this case report emphasizes the importance of a whole‐genome DNA copy number assessment in patients suspected for OI, which will elucidate the presence of precise *COL1A1* deletions and any pathogenic secondary copy number variations.

## Graphical abstract

Whole‐genome DNA copy number assessment in patients suspected for OI will elucidate the presence of precise *COLIA1* deletions and any secondary pathogenic copy number variations. This case report highlights the importance of large *COLIA1* deletions and secondary copy number variations in identifying clinical features which may establish as yet unknown variants of OI and lead to improved management and genetic counseling of patients.

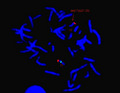

## 1 INTRODUCTION

Osteogenesis imperfecta (OI) is a rare group of inherited genetic disorder characterized by increased susceptibility to fractures caused by increased bone fragility. OI is classified based on clinical, radiologic, and genetic features into at least 16 types (OI types I‐XIX) (Beary & Chines, 2020). OI is most commonly inherited in an autosomal dominant fashion, but there have also been reports of recessive forms (Christiansen et al., 2010; Marini, Cabral, Barnes, & Chang, 2014; van Dijk et al., 2009). About 90% of cases are due to mutations in *COL1A1* (OMIM 120150) or *COL1A2* (OMIM 120160) that result in quantitative or qualitative defects in type I collagen, a key structural constituent of the extracellular matrix of bone, tendon, ligament, and sclera. In contrast to mutations, whole gene deletion of *COL1A1* is a rare cause of OI, with only a few reports so far in the English language literature (Bardai et al., 2016; Jewell et al., 2017; van Dijk et al., 2010). A minority of cases of OI are caused by mutations in genes involved in either the posttranslational modification of type I collagen (e.g., *FKBP10* [OMIM 607063] (Maghami et al., 2018); *CRTAP* [OMIM 605497] (Marini et al., 2014); *LEPRE1* [OMIM 610339] (Marini et al., 2014); *PPIB* [OMIM 123841] (van Dijk et al., 2009) or in bone synthesis and homeostasis (e.g., *SERPINH1* [OMIM 600943] (Christiansen et al., 2010); *SP7* [OMIM 606633] (Lapunzina et al., 2010); *WNT1* [OMIM 164820] (Fahiminiya et al., 2013).

OI manifests with a wide range of phenotypic expression and severity, from premature adult‐onset osteoporosis (OI type I) to multiple fractures in utero and perinatal death (OI type IIA) (Sillence, Senn, & D. and Danks DM., 1979) OI type I is a mild form characterized by fractures from minor trauma, mild, if any, skeletal deformities, blue‐colored sclera, conductive and sensorineural hearing impairment, and severe early onset osteoporosis. Dentinogenesis imperfecta (weak, discolored, and translucent teeth) is uncommon in OI type I, but is seen more frequently in other types like OI types II, III, IX, and X (Beary & Chines, 2020). OI is easily diagnosed in individuals with clear‐cut clinical features and positive family history. In less evident cases, sequencing of *COL1A1* and *COL1A2* is recommended (van Dijk et al., 2012). mRNA or cDNA analysis or quantitative or qualitative analysis of type I collagen from cultured dermal fibroblasts can be performed when sequencing is unobtainable (van Dijk et al., 2012).

Management is mostly symptomatic and supportive, aiming to maintain as near normal overall physical condition as possible by, among other measures, avoiding physical trauma and reducing the frequency of fractures. To this end, physical therapy, modulation of physical exertion, and bisphosphonates (Rauch & Glorieux, 2004) have been used. Surgical intervention might be needed for fracture repair and correction of deformities. Families and caregivers also benefit from supportive care and genetic counseling.

## 2 CASE REPORT

The proband is a white female, the product of a non‐consanguineous union of a 21‐year‐old mother and 25‐year‐old father. A routine prenatal ultrasonogram at 5 months gestational age revealed a fracture of the right femur. A follow‐up prenatal ultrasonogram, and a radiograph at birth showed fracture resolution with mild symmetrical curvature of both femurs. The mother received routine prenatal care and multivitamin supplementation. Delivery was by elective cesarean section at 38 weeks gestational age due to concerns for the occurrence of fractures if vaginal delivery was attempted. The patient was born without complications with a birth weight of 2,722 grams (27th percentile). The patient was fed with adequate quantities of formula, then transitioned to solid foods, but without vitamin supplementation. Her postnatal weight gain was appropriate, though linear growth was slow with length percentiles below the 5th percentile. She achieved all developmental milestones when due. She received all immunizations as scheduled.

At 3 months of age, the patient was brought to the emergency department by her parents with a 3‐day history of excessive inconsolable crying, discomfort, and inadequate oral intake. Examination revealed crepitus at the left rib cage and blue sclera. X‐rays showed non‐displaced fractures of the 6th–8th left ribs. The parents denied trauma, and there were neither physical signs of child abuse nor neurocutaneous markers of syndromic/congenital disease. While carrying out a peripheral venous blood draw, a nurse heard a cracking sound followed by crying and signs of distress. Radiographs revealed a non‐displaced, transverse, right proximal ulnar fracture (Figure S1). The patient received in‐patient therapy with analgesics and splints to the arm and made a full recovery.

At 6 months of age, she presented to her primary care physician for evaluation of left lower extremity pain. The possibility of a hitherto undetected healing left femur fracture was considered, but imaging was not obtained. Type I OI was thus suspected, and the patient was referred to our care for confirmation of the diagnosis and further management.

On initial presentation at our hospital at 10 months of age, the patient had a small stature with height and weight in the 2nd and 37th percentiles, respectively. Her sclera was blue, and there was mild bowing of the left femur, but her teeth appeared normal, without evidence of dentinogenesis imperfecta (discolored, weak teeth, sometimes associated with OI). Radiographs revealed marked under‐mineralization of bones but no vertebral compression abnormalities. Sequencing and copy number variation of *COL1A1* and *COL1A2* performed at an outside institution identified a heterozygous deletion of at least 18.5 kb, including *COL1A1*. On a subsequent follow‐up visit, the parents reported a non‐traumatic right fibula fracture that occurred at 18 months of age.

Family history is notable for a reportof four to five lifetime fractures in the father, who also has blue/gray sclerae and a normal stature. Copy number variation analysis done at an outside institution confirmed the father to be a carrier of the heterozygous deletion in *COL1A1* reported as being at least 14.5 kb in size. The mother's medical and family histories were non‐contributory. The patient's infant female sibling is reported by the parents to have blue sclerae but has not had fractures to date.

Dietary counseling was provided with a focus on ensuring adequate calcium intake and vitamin D supplementation. The parents were educated about the nature of the disease and preventive measures against trauma. aCGH was carried out on all members of the family; findings in the proband were confirmed with fluorescence in situ hybridization (FISH).

## 3 Methods

### 3.1 Ethical compliance

This case report was approved by the Research Subjects Review Board (RSRB) University of Rochester.

### 3.2 aCGH and FISH analyses

Microarray comparative genomic hybridization (aCGH) experiment was carried out on Agilent's SurePrint G3 ISCA CGH+SNP Microarray Kit, 4 × 180K v2.0 platform (Agilent Technologies). Briefly, the patient's DNA and control DNA (1.0 µg) were digested with restriction enzymes, Alu1 and Rsa1, and enzymatically labeled with dyes Cyanine‐5dUTP and Cyanine‐3dUTP, respectively, using Sure Tag DNA labeling kit (Cat # 5190–3399) as per the manufacturer's recommendations. The labeled DNA was hybridized as per the manufacturer's recommendations at 65°C for 40 hr. Data analysis was performed post‐washing. The slides were scanned in a high‐resolution scanner (Model # G2505C; Agilent Technologies Inc.) at 3‐µm resolution. The output files were processed and analyzed by CytoGenomics software v2.5 (Agilent Technologies Inc.), A log2 ratio values of less than −0.25 are scored as “LOSS” while oligos exhibiting values greater than +0.25 are scored as “GAIN.”

FISH confirmation of the aCGH findings was performed by the bacterial artificial chromosome (BAC) probes RP11‐262B3 (8p21.3;SO)/TelVysion 8p (SG), SureFISH CTNNA3 10q21.3;SG)/TelVysion 10q (SO); and RP11‐911N6 (17q21.33;SG)/TelVysion 17q (SO), respectively, using nick translation kit (Cat # 07J00‐001, Abbott Laboratories) according to manufacturer's instructions. Hybridization was performed per the manufacturer's instructions and standard protocols. The slides were analyzed using a Nikon (Eclipse 80i) fluorescence microscope attached with a CCD camera; Cytovision FISH software was used for image acquisition and analyses. Ten metaphases and 100 interphase cells were analyzed for confirmation of aCGH findings.

## 4 RESULTS

**Figure 1:**
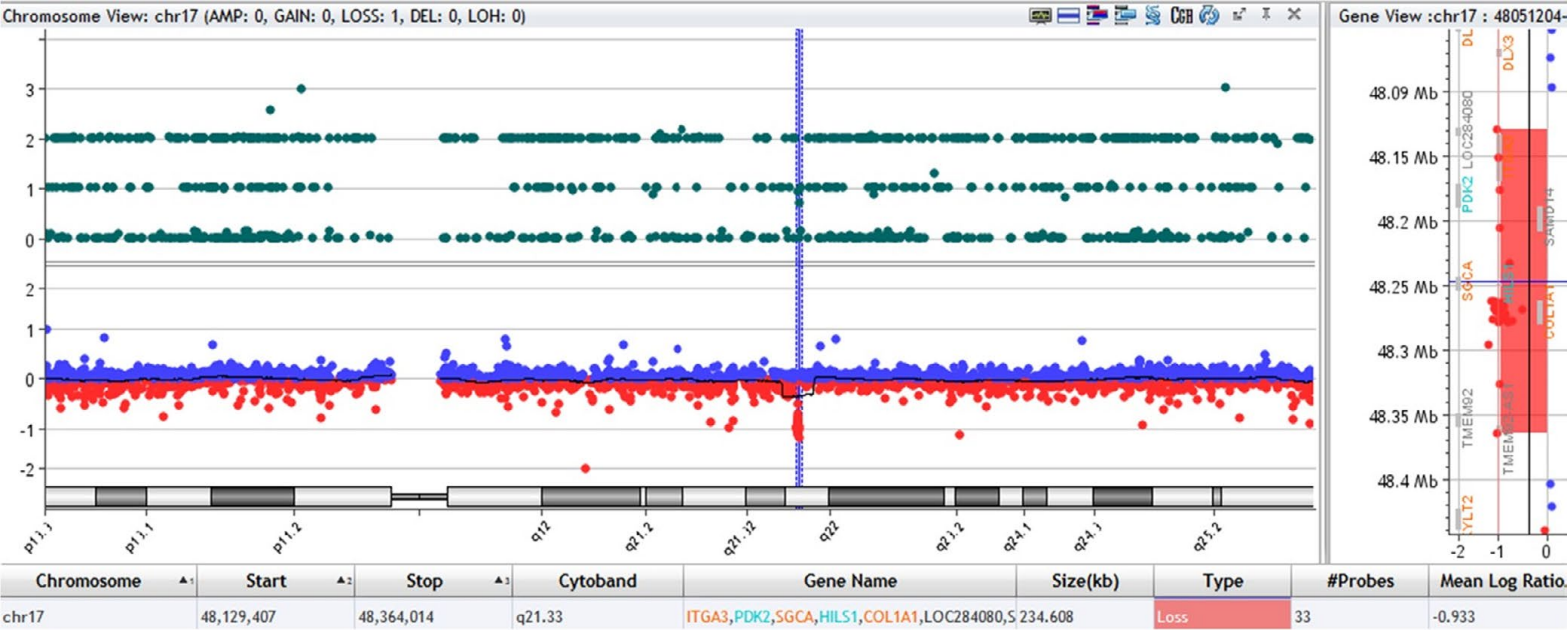
Chromosome view, gene view, and interval table, microarray comparative genomic hybridization (aCGH) of proband showing a 235 kb deletion at 17q21.33 from 48,129,407 to 48,364,014 base pairs encompassing *COL1A1*,* ITGA3*,* PDK2*,* SGCA*, and *HILS1*. The deletion is virtually identical to that seen in the father

**Figure 2:**
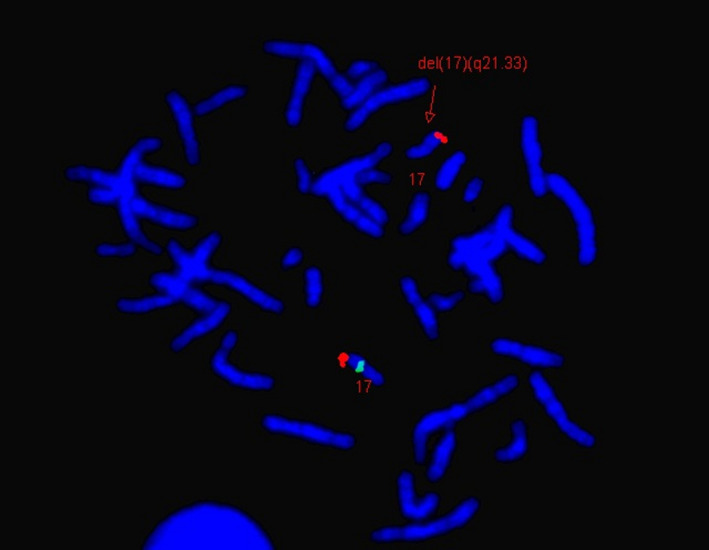
Confirmation of del (17) (q21.33) in the proband by FISH using RP11‐911N6 (17q21.33; Spectrum Green) target and TelVysion 17q (Spectrum Orange) control probes

A 235 kb deletion at 17q21.33 encompassing *COL1A1* and contiguous genes—*ITGA3* (OMIM 605025), *PDK2* (OMIM 602525), *SGCA* (OMIM 600119), and *HILS1* (OMIM 608101) (Figure 1)—was detected in the proband. The proband also harbors two other copy number variants—a 163 kb deletion at 10q21.3 involving *CTNNA3* (OMIM 607667) (Figure S2); and a 283 kb duplication at 8p21.3 involving *CSGALNACT1* (OMIM 616615) (data not shown). FISH was performed to confirm the 17q21.33 deletion in the proband (Figure 2). Follow‐up parental microarray confirmed paternal inheritance of the *COL1A1* deletion. The mother is negative for the 17q21.33 deletion but has the other two copy number variants del 10q21.3 and dup 8p21.3 found in the proband. The sibling harbors the same copy number variants (del 10q21.3 and dup 8p21.3) seen in the mother and the proband.

## 5 DISCUSSION

Whole gene deletion of *COL1A1* is a rare cause of OI type I (Bardai et al., 2016; van Dijk et al., 2010). The proband's *COL1A1* deletion is paternally inherited. The occurrence of relatively few lifetime fractures (4–5) in the father, coupled with his normal stature is evidence of a mild clinical phenotype. This is in agreement with other studies that have also found that complete *COL1A1* deletion results in a clinical phenotype of mild osteogenesis imperfecta with increased susceptibility to fractures, variable short stature, blue sclerae, and minor, if any, skeletal deformities (Jewell et al., 2017; van Dijk et al., 2010). It is expected that the proband will have a similar mild course.

The infant sibling is healthy, with neither *COL1A1* deletion nor features of osteogenesis imperfecta. Blue sclera, as reported in the sibling, is a normal and frequent finding in infancy and, by itself, is not evidence of disease. In infancy, the usually thin and transparent sclera enables the darker color of the underlying uvea to show through, thus imparting a blue hue to the sclera (Remington, 2012).

Disease phenotype may be modified by deletion of genes contiguous with the causative gene deletion (Bardai et al., 2016; Harbuz et al., 2013). We are still studying the clinical impact, if any, in the proband of deletions of the genes adjoining *COL1A1* (*ITGA3*,* PDK2*,* SGCA*,* HILS1*).

*ITGA3* (Integrin Subunit Alpha 3) encodes the integrin alpha cell surface adhesion molecule. The encoded alpha 3 subunit links non‐covalently with a beta 1 subunit to form Alpha‐3‐beta‐1 integrin. Alpha‐3‐beta‐1 integrin has been shown in animal models to be involved in the regulation of migration and layer formation in cerebral cortical neurons (Dulabon et al., 2000). Published reports of intellectual disability in patients with deletion of *COL1A1* and adjacent genes, including *ITGA3* (Jewell et al., 2017), elevate our concern, but to date, the proband's (and sibling's) intellectual and behavioral development have been within normal limits. The mother is also of normal intelligence.

The *SGCA* (alpha‐sarcoglycan) gene encodes α‐sarcoglycan (formerly known as adhalin), a protein that, among other functions, serves to maintain muscle fiber membranes during contraction. *SGCA* mutations cause autosomal recessive limb‐girdle muscular dystrophy‐3 (LGMDR3) (Al‐Harbi & Abdallah, 2016; Gonzalez‐Quereda et al., 2018), formerly known as limb‐girdle muscular dystrophy type 2D (Straub et al., 2018). GMDR3 is a disease with varying age of onset, severity, and clinical features, most prominently proximal muscle weakness and wasting (Angelini et al., 1998). Other features seen in some cases include heart failure from cardiac muscle involvement and respiratory failure from respiratory muscle weakness (Angelini et al., 1998). So far, the proband is without evidence of skeletal or cardiac muscle compromise. Also, the father, who is now in the latter part of the third decade of life, is without symptoms of muscle weakness. Furthermore, there is no family history of neuromuscular disorders.

*CTNNA3* is an adhesion molecule that is highly expressed in the heart. Deletion of the *CTNNA3* gene has been associated with cardiac abnormalities (Li, Goossens, & Hengel, 2012). An ongoing close cardiac evaluation has been unrevealing except for a clinically insignificant minimal pericardial effusion found on echocardiographic surveillance. The mother and infant sibling, both of whom carry an identical deletion, do not show any symptoms of cardiac disease. The proband is still undergoing close clinical follow‐up. Thus far, we have found no convincing evidence in the published literature that germline duplication of 8p21.3 involving *CSGALNACT1* is anything other than an incidental finding.

This study provides further support for the utility of aCGH in the molecular diagnosis of patients with suspected type I OI (Jewell et al., 2017). With more widespread incorporation of aCGH into the diagnostic armamentarium, more cases of OI due to complete gene deletion will likely be identified. Importantly, as illustrated in this case, aCGH allows for more complete characterization of molecular defects due to copy number variations than what can be identified on targeted single gene or gene panel analysis. Together with previously reported cases of deletions, we recommend that the presence of such large deletions and secondary copy number variations be considered for identification of clinical features which may establish as yet unknown variants of OI and lead to improved management and genetic counseling of patients.

## CONFLICT OF INTEREST

The authors have no conflict of interest to declare.

## AUTHORS CONTRIBUTION

NN wrote the manuscript with clinical input from DRW and GAP Jr. MAI supervised the DNA microarray analysis, interpreted the data, and reviewed the final draft of manuscript. All authors read and approved the final manuscript.

## Supporting information

Fig S1

Fig S2
