# Supplementary figures and images for: A 235 Kb deletion at 17q21.33 encompassing the COL1A1, and two additional secondary copy number variants in an infant with type I osteogenesis imperfecta: A rare case report
Source: Mol Genet Genomic Med. 2020 Apr 13;8(6):e1241. doi: 10.1002/mgg3.1241 (PMC7284024; doi:10.1002/mgg3.1241)

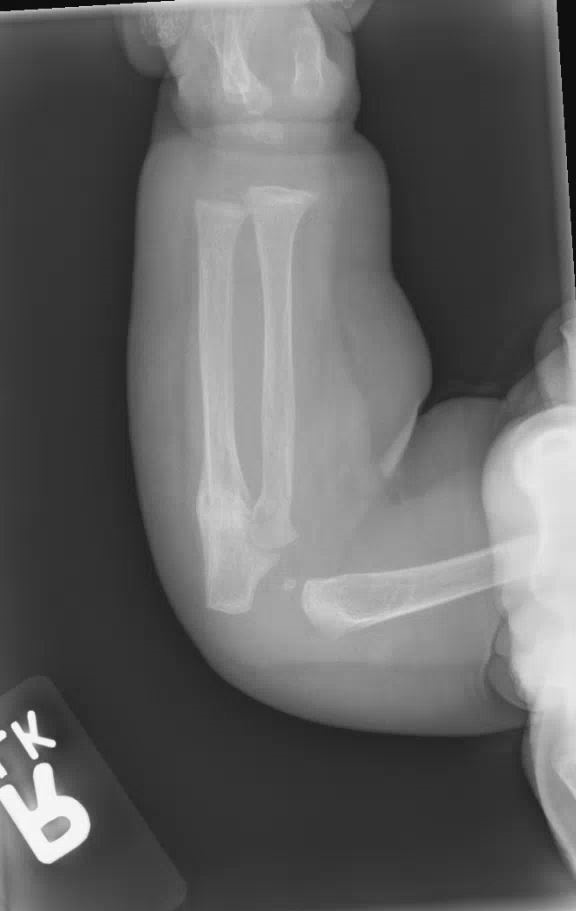

Supplement: Supplementary file 1 — Fig S1 [file MGG3-8-e1241-s001.tif]

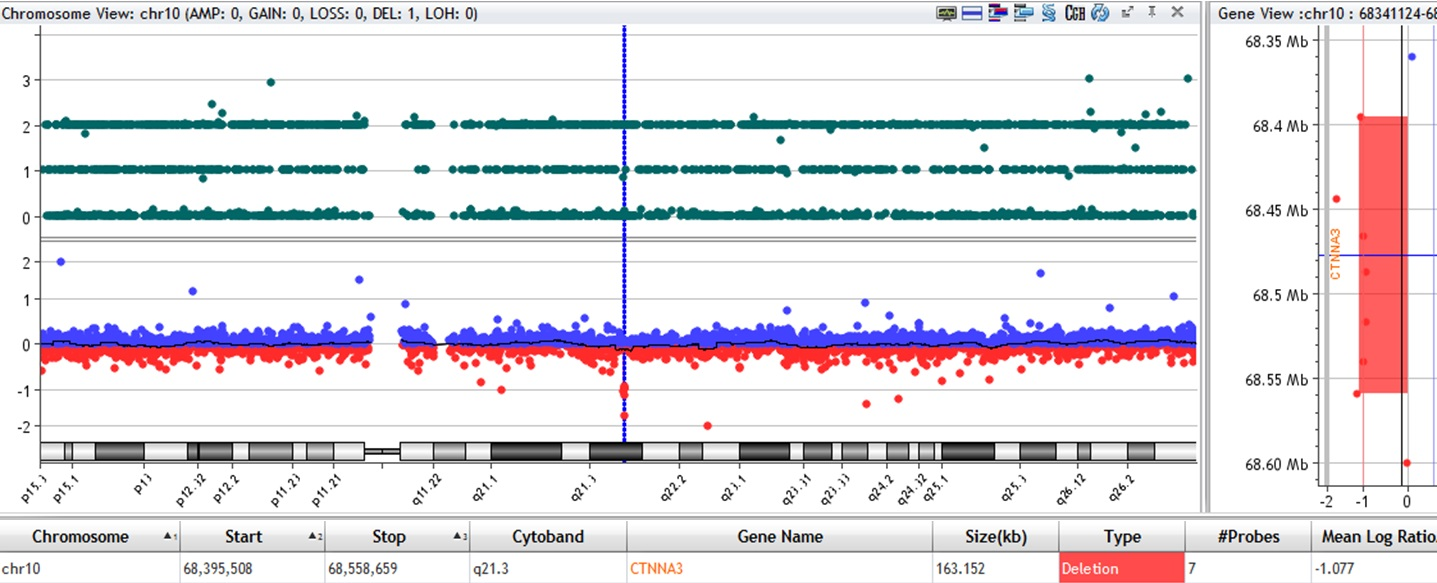

Supplement: Supplementary file 2 — Fig S2 [file MGG3-8-e1241-s002.tif]
